# Infection with the Neorickettsial Organism *Stellantchasmus falcatus* Agent in an Arctic Fox (*Vulpes lagopus*)

**DOI:** 10.1111/vcp.70109

**Published:** 2026-05-04

**Authors:** Jeremy P. Bessett, Alexis G. Davidson, Alyssa M. Scagnelli, Kristen R. Friedrichs

**Affiliations:** ^1^ Department of Pathobiological Sciences, School of Veterinary Medicine University of Wisconsin‐Madison Madison Wisconsin USA; ^2^ Department of Surgical Sciences, School of Veterinary Medicine University of Wisconsin‐Madison Madison Wisconsin USA

**Keywords:** blood, fox, *Nanophyetus salmincola*, PCR, rickettsia

## Abstract

A 1‐year‐old female‐intact arctic fox (*Vulpes lagopus*) presented for diarrhea, lethargy, severe thrombocytopenia, and hyperbilirubinemia. Blood film evaluation revealed intracytoplasmic coccoid bacteria within moderate numbers of leukocytes consistent with infection by a rickettsial organism. Similar organisms were observed within many leukocytes from fine‐needle aspirates of an enlarged peripheral lymph node. The lymph node also contained moderate necrosis and an expansion of granular lymphocytes. Parasitic ovum consistent with the trematode *Nanophyetus salmincola* was found on fecal floatation. The patient responded to treatment with doxycycline, enrofloxacin, ampicillin/sulbactam, and other supportive therapies. Polymerase chain reaction testing confirmed the presence of Neorickettsia sp., and DNA sequencing identified the organism as *Stellantchasmus falcatus* agent. To the authors' knowledge, this is the first report describing this organism microscopically in peripheral blood, the first reported case of infection in this species of canid, and describes the various clinicopathologic changes with this infection.

An approximately 1‐year‐old female intact arctic fox ( *Vulpes lagopus* ) was referred for a 1‐day history of diarrhea, lethargy, severe thrombocytopenia, and hyperbilirubinemia. Relevant clinical history for this patient included recent residence in the state of Oregon, where the patient was found roaming in an urban area. Since this species is not native to the Pacific Northwest region of the United States, it was presumed that the arctic fox was an escaped pet. The animal was first taken to a local rehabilitation center, followed by a zoological institution, where a physical exam revealed a thin body condition (2/9), but otherwise the patient appeared healthy. Radiographs and limited bloodwork were unremarkable, and fecal flotation was reportedly negative. The fox received selamectin topically as part of standard institutional quarantine protocols. A month later, the animal was transferred to a new facility. Three to four days after arriving at the new facility, diarrhea was noted. At that time, the referring veterinarian performed patient‐side fecal ELISA tests for canine parvovirus (SNAP Parvo, IDEXX Laboratories, Westbrook, ME, USA) and Giardia antigens (SNAP Giardia, IDEXX Laboratories, Westbrook, ME, USA), both of which were reportedly negative. Additionally, an in‐house fecal flotation was performed, and no ova or parasites were observed. Over a few days, lethargy, weight loss, and a marked decline in appetite were reported by staff at the new institution. The fox was evaluated by the referring veterinarian, and physical exam revealed mild hyperthermia (103.5F), prominent popliteal lymph nodes, and diffuse moderate muscle wasting. Abnormalities on a CBC and biochemical profile performed at the referring veterinary clinic included leukopenia, neutropenia, thrombocytopenia, azotemia, hyperphosphatemia, hypocalcemia, and increased ALT and ALP activities, and hyperbilirubinemia (Table 1) when compared to previously reported reference intervals for this species [1]. Fine needle aspirate of the left popliteal lymph node was evaluated in‐house by the referring veterinarian and was reportedly consistent with reactive lymphoid hyperplasia. Subcutaneous fluids, vitamin B injection, mirtazapine, maropitant, and metronidazole were administered and the fox was subsequently transferred to the referral institution the next day.

**TABLE 1:** Hematologic, serum biochemical, and coagulation assay data.

Analyte	10/12^a^	11/25^b^	11/26^c^	12/5^d^	Units
PCV	36	49	46	31	%
Total protein (Refractometry)	6.2	6.2	6.1	6.2	g/dL
RBC	—	8.91	8.43	6.08	×10^6^/μL
HGB	—	15.5	14.8	10.4	g/dL
HCT	—	44	48	32	%
MCV	—	49.4	56	54	fL
MCH	—	17.4	18	17	pg
MCHC	—	35.2	31	32	g/dL
PLT	—	26	11	138	×10^3^/μL
MPV	—	17.5	9.4	7.4	fL
WBC	—	2.7	2.22	3.75	×10^3^/μL
Neutrophils	—	0.85 (32%)	1.75 (79%)	2.17 (58%)	×10^3^/μL
Band neutrophils	—	Suspected	0.11 (5%)	0.0	×10^3^/μL
Lymphocytes	—	1.1 (41%)	0.18 (8%)	1.13 (30%)	×10^3^/μL
Monocytes	—	0.74 (27%)	0.11 (5%)	0.19 (5%)	×10^3^/μL
Eosinophils	—	0	0.04 (2%)	0.04 (1%)	×10^3^/μL
nRBC	—	—	0.02 (1%)	0.23 (6%)	×10^3^/μL
Reticulocytes	—	0.004	0.007	0.175	×10^6^/μL
ALP	—	1723	1745	350	U/L
GGT	—	6	21	—	U/L
ALT	—	133	189	321	U/L
AST	—	—	487	—	U/L
CK	—	—	844	—	U/L
T bili	—	4.0	6.0	0.6	mg/dL
BUN	—	81	74	36	mg/dL
Creatinine	—	1.3	0.9	0.8	mg/dL
BUN:Creatinine	—	62	82	45	
Total calcium	—	7.8	8.3	11.3	mg/dL
Phosphorus	—	10.1	7.8	9.7	mg/dL
Total protein (Biuret)	—	5.4	5.3	5.6	g/dL
Albumin	—	2.6	2.3	2.2	g/dL
Globulin	—	2.8	3.0	3.4	g/dL
Glucose	—	154	139	126	mg/dL
Cholesterol	—	207	207	—	mg/dL
Sodium	—	142	138	143	mmol/L
Chloride	—	101	114	—	mmol/L
Potassium	—	3.7	2.9	5.1	mmol/L
TCO_2_	—	—	8	—	mmol/L
Anion gap	—	—	19	—	
PT	—	—	10.4	—	Seconds
aPTT	—	—	32	—	Seconds

^a^ Initial Zoologic Institution.

^b^ Referring veterinarian blood work Procyte Dx and Catalyste One (IDEXX Laboratories, Westbrook, ME, USA).

^c^ University of Wisconsin Veterinary Care (UWVC) presentation blood work Advia 2120i, Vitros 4600, Stago Compact Max.

^d^ UWVC recheck blood work Advia 2120i, Vetscan VS2 (Abaxis, Union City, CA, USA).

## 1 Case Presentation

On presentation, the fox was normothermic (101.7°F) with tacky mucous membranes, decreased skin turgor, and sunken eyes with dehydration estimated at 10%. The animal had prominent popliteal lymph nodes, and the mucus membranes were icteric. Additionally, the fox was emaciated with severe diffuse muscle atrophy and fecal staining around the perineum.

Abdominal ultrasound was performed and revealed multiple subjectively mildly to moderately enlarged lymph nodes, the largest at the root of the mesentery, which measured 1.05 cm in width. There was subjective thickening of the colon with poorly discernable wall layering. The mucosal layer of the jejunum was subjectively thickened with perpendicular hyperechoic striations and minimal propulsive contractions. There was also hyperechoic suspended luminal material in the gallbladder, and scant hyperechoic peritoneal free fluid. The clinically relevant interpretation of ultrasonographic findings included generalized functional ileus, enterocolitis with mild lacteal dilation, scant peritoneal effusion, reactive lymphadenopathy, and biliary sludge.

A CBC was performed on the Advia 2120i (Siemens Healthcare Diagnostics, Tarrytown, NY, USA), serum biochemistry profile was performed on the Vitros 4600 (Ortho Clinical Diagnostics Inc., Rochester, USA), and prothrombin time (PT) and activated partial thromboplastin time (aPTT) were performed on the Stago Compact Max (Diagnostica Stago Inc., Parsippany, NJ, USA) (Table 1).

Routine blood film evaluation was performed by a medical technologist and flagged for pathologist review due to the presence of suspected microorganisms. Moderate numbers of leukocytes contained variable numbers of small, gray‐blue, irregular coccoid bacteria that formed round aggregates (morulae) or were dispersed throughout the cytoplasm. These inclusions were morphologically consistent with rickettsial microorganisms and were found in all leukocyte types, primarily monocytes, macrophages, neutrophils, and less commonly in lymphocytes and eosinophils (Figure 1). Low numbers of macrophages were observed at the feathered edge of the blood film that rarely contained small bile casts, hemosiderin, and/or phagocytosed erythrocytes. Due to the dispersion of microorganisms and presence in all leukocyte types, infection with *Anaplasma phagocytophilum* , *Ehrlichia canis* , or *Ehrlichia chaffeensis* was considered unlikely.

**FIGURE 1:**
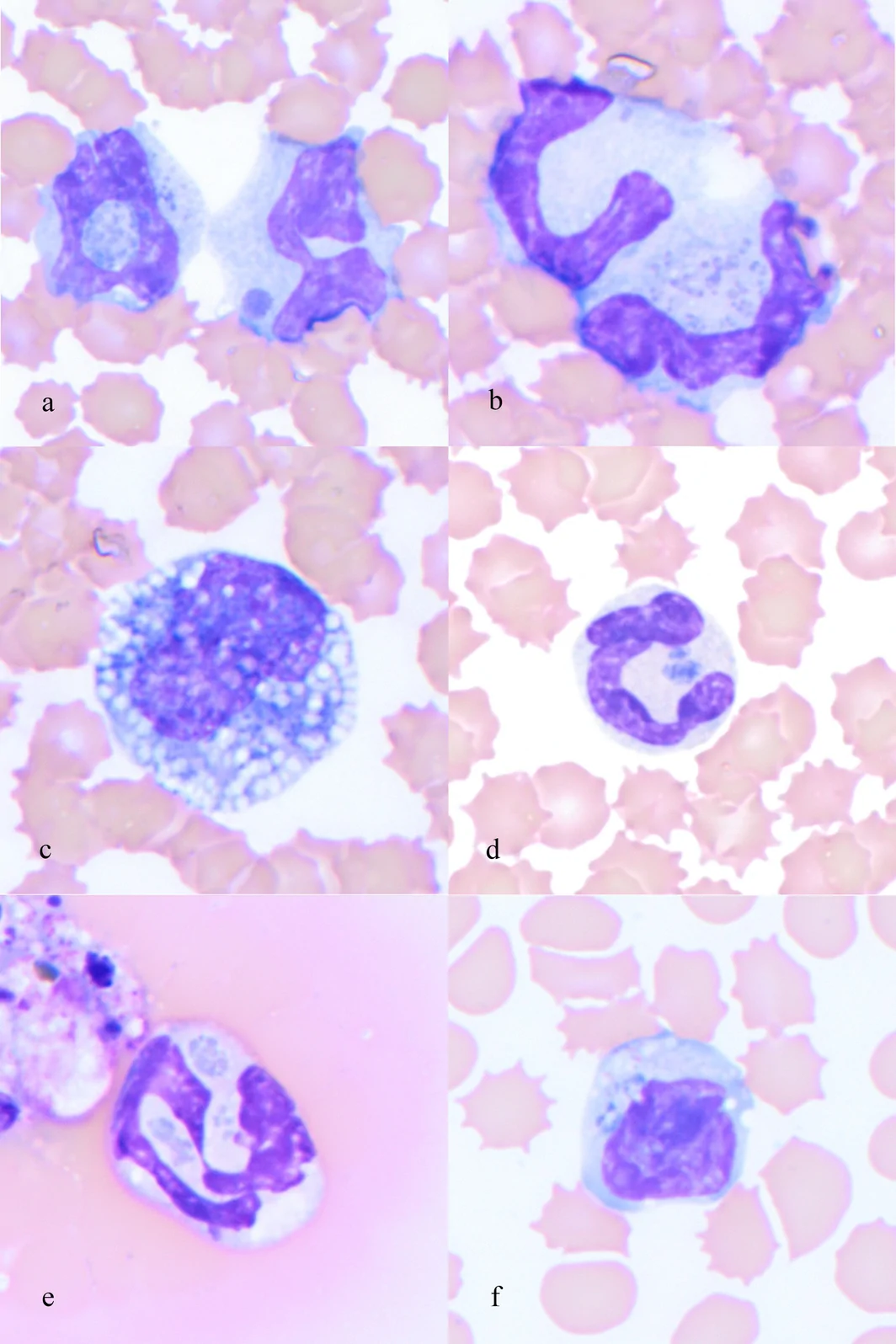
Peripheral blood film from an Arctic fox. Leukocytes containing the SF agent microorganism inclusions. Monocytes (a, b), macrophages (c), neutrophils (d, e), and lymphocytes (f). Modified Wright–Giemsa stain. Original image 100× objective.

Fine‐needle aspirates of the enlarged left popliteal lymph node were obtained and routinely stained (Modified Wright–Giemsa stain, Aerospray, Wescor Inc.). The preparations were of low to moderate cellularity and contained moderate amounts of necrotic cellular debris in a mildly bloody background. A heterogeneous population of small and large lymphocytes predominated, with a mildly to moderately increased proportion of intermediate to large granular lymphocytes. Other nucleated cells included mildly increased numbers of macrophages and non‐degenerate neutrophils. Macrophages were individually identified, variably vacuolated, and often contained phagocytic debris, including nuclear material, erythrocytes, and less often, hemosiderin. Many of the leukocytes within the lymph node contained microorganisms morphologically similar to those observed in peripheral blood. Unlike the peripheral blood, where microorganisms were predominantly found within myeloid lineage cells, microorganisms were primarily found within lymphocytes in the lymph node (Figure 2). Rarely, small aggregates of uniform spindle cells were observed and interpreted to be either reactive fibroblasts or endothelium. No rickettsial organisms were observed within the spindle cell population. A rare, disrupted neutrophil appeared to contain intracellular, distinct, dark purple cocci bacteria. The cytologic interpretation of the lymph node sample was reactive lymphoid hyperplasia with large granular lymphocyte expansion, marked necrosis, numerous predominantly intracytoplasmic rickettsial organisms, and neutrophilic and macrophagic inflammation with rare cocci bacteria.

**FIGURE 2:**
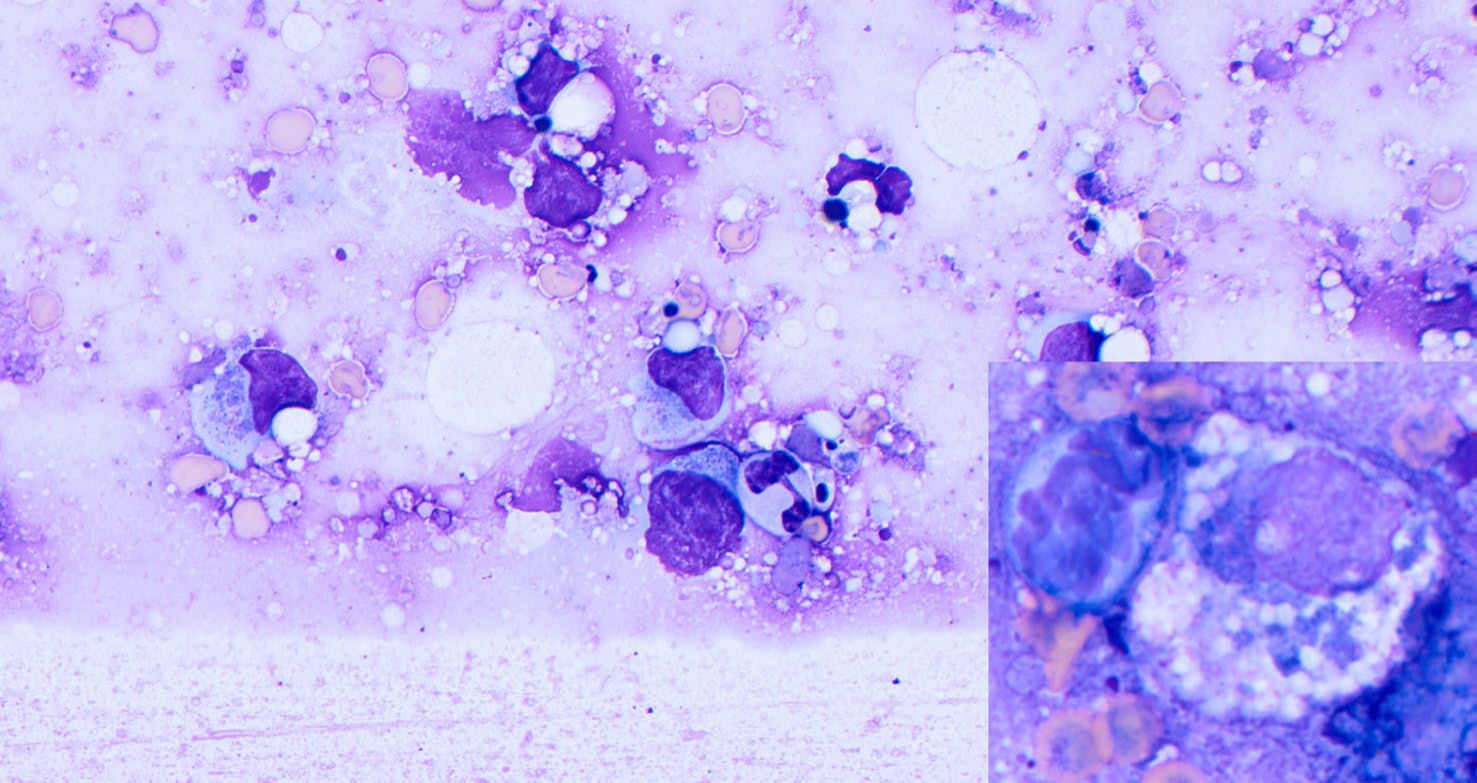
Lymph node from an arctic fox. Lymphocytes and macrophages (inset) containing the SF agent microorganism inclusions. Modified Wright–Giemsa stain. 60× objective, original inset image 100× objective.

Fecal floatation was performed via zinc sulfate centrifugation, yielding moderate numbers of ovoid yellow‐brown parasitic ova that averaged 80 × 49 μm with a thin, clear wall, an indistinct operculum at one pole, and often a small knob‐like structure at the opposite pole. Ova had a granular internal appearance that occasionally had a “turtle‐shell‐like” appearance. Ovum were interpreted to be morphologically consistent with *Nanophyetus salmincola* (Figure 3).

**FIGURE 3:**
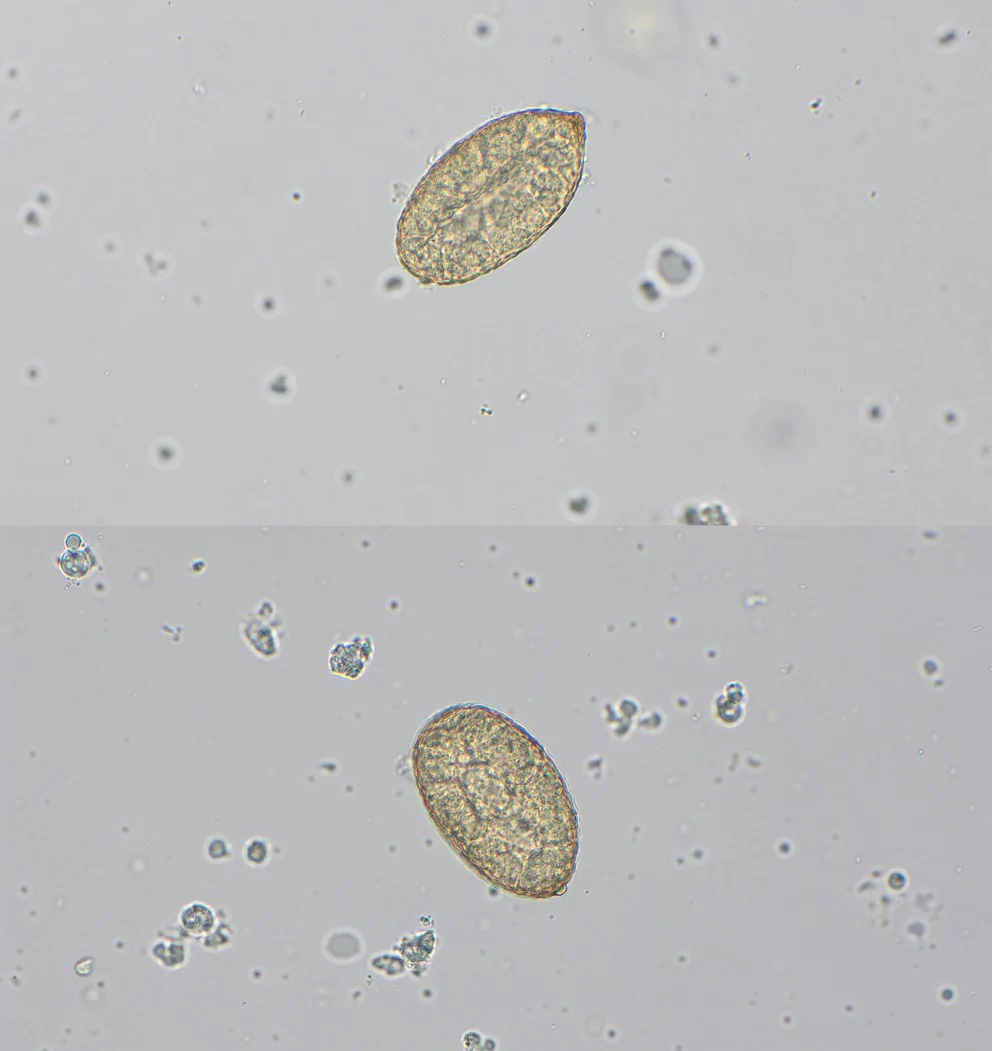
Fecal float (zinc sulfate) from an arctic fox. *Nanophyetus salmincola* ova. Unstained wet mount. Original image 40× objective.

Serum was submitted for Leptospira MAT titers; all were reported as negative (< 1:100). Serum and EDTA whole blood samples were submitted to the North Carolina State University Tick and Vector‐Borne Disease Laboratory. Antibody testing for *Ehrlichia canis, Ehrlichia ewingii, Anaplasma phagocytophilum, Anaplasma platys*, and *Borrelia burgdorferi*, and antigen testing for *Dirofilaria* was performed (SNAP 4Dx plus, IDEXX Laboratories) and were negative. Additionally, serum was used to detect antibodies by immunofluorescent assay (IFA) to *Bartonella* spp., *Ehrlichia canis* , and *Babesia canis*, and via ELISA to *Babesia gibsonii*; all were negative. Whole blood was used to perform PCR testing for *Anaplasma* genus, *Anaplasma platys* , *Anaplasma phagocytophilum* , *Babesia* genus, *Apicomplexa*, *Bartonella* genus, *Ehrlichia* genus, *Hemotropic Mycoplasma* genus, *Rickettsi*a genus, and *Neorickettsia* genus. The *Neorickettsia* genus PCR produced a positive result. DNA sequencing identified an organism with 99% similarity to *Neorickettsia* sp. *SF* (also known as *Stellantchasmus falcatus* agent).

Pending antibody, PCR, and fecal results, the patient was hospitalized for monitoring and supportive care. She initially received intravenous fluids (70 mL/kg/day), metoclopramide (CRI at 2 mg/kg/day), maropitant (1 mg/kg IV q24h), ondansetron (0.5 mg/kg IV q8h), Unasyn (22 mg/kg IV q8h), doxycycline (5 mg/kg PO q12h), and enrofloxacin (10 mg/kg IV q24h). The next day, she was still dehydrated, so intravenous fluids were increased (100–150 mL/kg/day). The patient became painful in her caudal abdomen, so analgesia via ketamine CRI was initiated (3–5 mcg/kg/min), which provided adequate pain control. All other treatments were continued. Upon discovery of *Nanophyetus salmincola* ova in the feces, praziquantel (20 mg/kg PO once) was administered. Over the next 48 h, the fox displayed an improved mentation and regained her appetite. Intravenous fluids were weaned, and the patient was discharged 4 days after admission to the hospital. The fox was discharged on S‐Adenosylmethionine (SAMe)/Silybin‐phosphatidylcholine complex (90/31 mg PO q24h) for 30 days, doxycycline for 28 days (5 mg/kg PO q12h), enrofloxacin for 10 days (10 mg/kg PO q24h), and amoxicillin/clavulanic acid for 10 days (13 mg/kg PO q12h). A CBC and biochemistry profile was repeated 6 days after discharge (about 9 days after initial presentation) (Table 1) and was overall interpreted to be markedly improved. The fox continued to improve and remains clinically well almost 1 year after diagnosis and successful treatment.

## 2 Discussion

*Stellantchasmus falcatus* is a small intestinal trematode that has been reported in Asian‐Pacific countries. The trematode has been described as infecting multiple species of fish and several species of mammals [2, 3, 4]. *Stellantchasmus falcatus* agent (SF agent) was first identified in Japan in 1962 from *Stellantchasmus falcatus* metacercaria, and at the time was classified as a member of the *Ehrlichieae* tribe, but it has since been reclassified as a species of *Neorickettsia* [4, 5]. A study in 1996 found through sequencing that the SF agent is most similar to *Neorickettsia risticii* , *Neorickettsia sennetsu* , and *Neorickettsia helminthoeca* with 99.1%, 98.7%, and 93.7% similarity, respectively [4].

In 1981, SF agent infection was first described experimentally in a dog in Japan. In this experiment, infection with the SF agent produced a fever and no other clinical signs [6]. In 1973, a Neorickettsial organism immunologically distinct from *N. helminthoeca* called Elokomin fluke fever agent (EFF agent) was identified in bears fed *Nanophyetus salmincola* infected trout in the United States. Samples from infected bear lymph nodes with microscopically confirmed rickettsial organisms were inoculated into dogs producing mild signs of illness including fever, lethargy, anorexia, vomiting, diarrhea, lymphadenomegaly, and occasionally mucopurulent ocular discharge and ataxia [7]. In 2017, molecular studies identified the SF agent within *N. salmincola* flukes in the United States [8]. It is unknown if the EFF agent and SF agent are the same or if the EEF agent is yet another distinct Neorickettsial organism similar to *N. helminthoeca*, since sequencing was not performed on the original EEF agent samples [7, 8]. The incubation period for the SF agent is not known currently. Clinical signs in one case were reported 10 days after inoculation with the SF agent [6]. It is possible that *N. salmincola* was contracted by this arctic fox while roaming in Oregon through the consumption of fish infected with this fluke, which then later led to SF agent infection. Another potential source of SF agent infection would be if this patient was fed raw fish infected with *N. salmincola* while at the original zoologic institution in Oregon.

In North America, naturally occurring infection with the SF agent was first described in three dogs from the Pacific Northwest of the United States in 2020. These patients were severely ill, failed to respond to conventional treatments, and subsequently expired. Histologic evaluation of necropsy samples identified granulomatous and necrotizing splenitis and lymphadenitis, severe histiocytic and lymphoplasmacytic enteritis, random piecemeal hepatocellular necrosis and degeneration, among other changes [9].

Infection with the SF agent has not been described in *Vulpes* spp. Additionally, hematologic features have not been described, and cytologic features have been only briefly described with this infection. Interestingly, in this case, microorganisms were observed in peripheral blood and did not display a clear tropism for specific leukocyte types. It is unknown to what effects, if any, previous treatment of this fox with metronidazole could have had on the morphology, intracellular distribution, or type of leukocytes infected. In the peripheral blood, most of the cells infected were monocytes, macrophages, and neutrophils; in the lymph node, lymphocytes were the most common cells infected. The cause of the difference observed is unknown, but it is possible that infected lymphocytes were relatively rare in peripheral blood because of the low circulating lymphocyte concentration. The mechanism for lymphopenia in this case is unclear, could be multifactorial, and may include the effects of cortisol and lymph node necrosis.

Cytologically, necrosis within the lymph node appears consistent with histologic observations previously described [9]. In our sample, macrophages and neutrophils appeared increased; however, compelling evidence of granulomatous inflammation was not observed in the sample. Macrophages were typically dispersed individually throughout the sample and did not appear to form multinucleate giant cells or sheets of epithelioid macrophages. Increases in neutrophils and macrophages were attributed primarily to necrosis, but some degree of granulomatous or pyogranulomatous inflammation cannot be ruled out as this has been observed with SF agent infections as well as with salmon poisoning disease. The observed large granular lymphocyte expansion was interpreted as a cytotoxic T‐lymphocyte and/or natural killer cell response to this intracellular infection. Unfortunately, due to the patient's marked thrombocytopenia, sampling of the enlarged mesenteric lymph nodes for cytologic or histologic examination could not be performed safely. Cytologic examination of additional peripheral lymph nodes might have been interesting to determine if the left popliteal lymph node findings were repeatable in other peripheral lymph nodes.

The presence of non‐rickettsial bacterial organisms within the lymph node was an unexpected finding. It is unclear if the neutrophil containing cocci bacteria represented a localized secondary bacterial infection in the lymph node or possibly sepsis in this patient. Sepsis could be further supported, given the severity of the patient's general clinical signs and neutropenia with a left shift and toxic change. Blood culture was considered, but due to patient demeanor and stress, empirical broad‐spectrum antimicrobial therapy was instituted instead.

Given that the blood work was analyzed on several different hematology and chemistry analyzers, it is challenging to compare results obtained by the referring veterinarian and the various time points at the referral institution. Additionally, interpretation of laboratory data is challenging in more unique exotic species, as it was here, due to the sparsity of publications and data regarding normal values and reference intervals. Despite this, most of the values of significant clinical concern had improved over time, including platelet, total bilirubin, and BUN concentrations, ALT and ALP activities, and BUN:Creatinine ratio.

Laboratory data was interpreted based on approximations to previously described reference intervals for this species, established reference intervals in similar fox species, clinician experience, and what is generally considered normal for mammalian species [1]. The moderate hyperbilirubinemia was mostly attributed to a cholestatic process in this patient given the markedly high alkaline phosphatase activity and mildly high GGT activity. In previous reports of this infection, hepatic necrosis was reported [9], and although sampling of the liver to evaluate for the presence of necrosis was not performed, some degree may be present given the presumed mild increases in ALT and AST activities. If sepsis was truly present in this patient, this could have also contributed to hyperbilirubinemia. The presence of macrophages containing bile casts in peripheral blood is unusual and the exact cause cannot be determined. However, the finding could be explained by a cholestatic hepatopathy, concurrent liver injury (e.g., necrosis), and recirculation of inflammatory macrophages from the injured liver tissue. The cause of low platelet concentration may be multifactorial. First, the magnitude of this patient's low platelet count suggests a possible component of immune‐mediated platelet destruction. Second, a consumptive component (e.g., disseminated intravascular coagulopathy) is also possible, and could be supported by the presumptive prolongation of PT and aPTT assays.

In summary, to the authors' knowledge, this is the first report of SF agent infection in an arctic fox and the first report of the observation of the SF agent in a peripheral blood film from any species. This organism appears morphologically similar to other Neorickettsial organisms, and this case highlights that species identification of these organisms requires molecular techniques (e.g., PCR). Lastly, this report details a positive clinical response following therapeutic intervention that has not been reported in other canine cases.

## Conflicts of Interest

The authors declare no conflicts of interest.
